# Detection of adulteration in Iranian grape molasses added glucose/fructose/sugar beet syrups with ^13^C/ ^12^C isotope ratio analysis method

**DOI:** 10.1002/fsn3.4259

**Published:** 2024-09-10

**Authors:** Vahid Jamali, Aryou Emamifar, Hadi Beiginejad, Mohammad Moradi, Mousa Rasouli

**Affiliations:** ^1^ Grape Processing and Preservation Department, Research Institute for Grapes and Raisin Malayer University Malayer Iran; ^2^ Department of Food Science and Technology, College of Food Industry Bu‐Ali Sina University Hamedan Iran; ^3^ Department of Horticultural Sciences Engineering, Faculty of Agriculture and Natural Resources Imam Khomeini International University Qazvin Iran

**Keywords:** adulteration, food fraud, grape molasses, isotope ratio

## Abstract

Grape molasses (GM), produced from grapes, is a traditional Iranian food and is widely consumed in Iran. However, GM adulteration is among the most widespread illegitimate procedures involving contamination of food with foreign materials, such as adding sugar–water solution, date syrup, sugar beet syrup, and grape sauce. This study used stable carbon ^13^C/^12^C isotope ratio analysis method to detect adulteration of GM samples with glucose syrups (GS), fructose syrups (FS), and beet sugar syrups (BS) at the ratio of 0%, 10%, 30%, and 50% (by weight). Physicochemical properties of GM including °Brix, conductivity, specific gravity, pH, moisture content, ash content, hydroxymethyl furfural, sugar content, and rheological properties of samples were investigated. The δ^13^C isotope ratio of the GM was determined as −26.61%, that of the GS as −13.23%, that of the FS as −13.42%, and that of the BS as −16.58%. The δ^13^C isotope ratio increased by the addition of adulterant syrups to GM. The addition of each adulterant syrup had a different effect on the physicochemical parameters; however, the °Brix and specific gravity had a positive correlation with the δ^13^C isotope ratio results. The magnitudes of G' and G" increase with an increase in frequency representing the viscoelastic behavior of samples. The obtained results of this study suggest the use of δ^13^C isotope ratio method as a fast and accurate method to investigate the adulteration of grape molasses.

## Graphical abstract

Grape molasses (GM), produced from grapes, is a traditional Iranian food and is widely consumed in Iran. However, GM adulteration is among the most widespread illegitimate procedures involving contamination of food with foreign materials, such as adding sugar–water solution, date syrup, sugar beet syrup, and grape sauce. In this paper, the performance of stable carbon ^13^C/^12^C isotope ratio analysis method as a fast technique to investigate adulteration in grape molasses was investigated. Grape molasses was adulterated with fructose, glucose, and sugar beet syrups and then adulteration was measured. This method had a positive (*R* ^2^ > .95) correlation with °Brix and specific gravity. The obtained results can be useful as a reliable method to find adulteration in grape molasses.

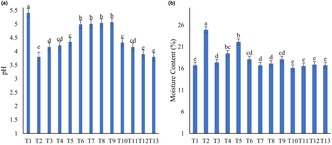

## 1 INTRODUCTION

Grape molasses (GM), a sweet product, is obtained from crushing, clarifying, and boiling the juice of some grape varieties that are not suitable for fresh consumption in the market. Unfermented grape juice is concentrated by heat processing without the addition of sugar or other food additives, and the resulting syrup is used as a sweet concentrated syrup with total soluble solids of 70%–80% in the food industry. It is also consumed as a dessert in some communities (Ghasemi‐Varnamkhasti et al., 2019; Heshmati et al., 2020).

The GM is a valuable energy and carbohydrate source due to its high content of natural sugars in the form of glucose, fructose, and galactose, minerals such as iron, phosphorus, calcium, magnesium, and potassium (Akan, 2018; Güçlü et al., 2023; Naderi‐Boldaji et al., 2018). GM has many uses in different food industries, such as some cookies, pastries, and dairy products. It can be ingested directly or used to sweeten sesame paste (Ardeh) consumed for breakfast (Güçlü et al., 2023; Naderi‐Boldaji et al., 2018).

Food adulteration refers to the intentional or unintentional addition of physical and chemical ingredients that are not normally present in the product. It is a universal concern worldwide not just because food adulteration is a fraud played on consumers, but it can also harm the health and cause serious outcomes to the well‐being of people (Naderi‐Boldaji et al., 2018; Razavi & Kenari, 2023). The adulteration in grape molasses is majorly performed by utilizing adulterants, such as corn syrup, inexpensive juices, sugar, and water, which use has been motivated by the associated economic gain (Jha et al., 2016).

Chemical techniques have been developed for the detection of food adulteration. The most widely used technique in detecting contaminated liquid food is the high‐performance liquid chromatography (HPLC), which however does not detect low levels of adulteration. The gas chromatography/mass spectroscopy (GC/MS) technique is a precise methodology that can detect low to high levels of adulteration, which is calculated by the ^13^C/^12^C isotope ratio. The different ratios of carbon isotopes are produced by different photosynthesis cycles (Çaçan et al., 2023). In previous studies, the efficiency of the ^13^C technique in identifying honey frauds has been proven. But it has not been applied to identify adulterated grape molasses (Geană et al., 2020; Li et al., 2024; Yücel et al., 2023).

Today, GM is used as a natural sweetener in many sugar‐free or low‐sugar food products. It is also recommended for anemic patients due to its high iron content. Therefore, considering the nutritional value of this product as well as the target group of consumers, it is very important that the product must not be adulterated. Although previous studies have investigated the adulteration of mulberry, carob, fig, and grape syrups using chemical and spectroscopic methods (Naderi‐Boldaji et al., 2018; Tosun, 2014), it is the first time that a study has chosen to evaluate the adulteration levels in grape molasses using the isotope ratio assay along with rheological behavior. The chemical composition of GM may change due to adulteration; therefore, it is necessary to investigate the physicochemical properties of grape molasses with a precise method.

## 2 MATERIALS AND METHODS

### 2.1 Materials

White seedless grapes (*Vitis vinifera* L.) were gathered from Malayer vineyards (Hamedan, Iran). Grapes were transferred to the laboratory and stored at 4°C. All chemicals were of analytical grade and were purchased from Merck & Co., Inc.

### 2.2 Methods

#### 2.2.1 Grape molasses production

**FIGURE 1:**
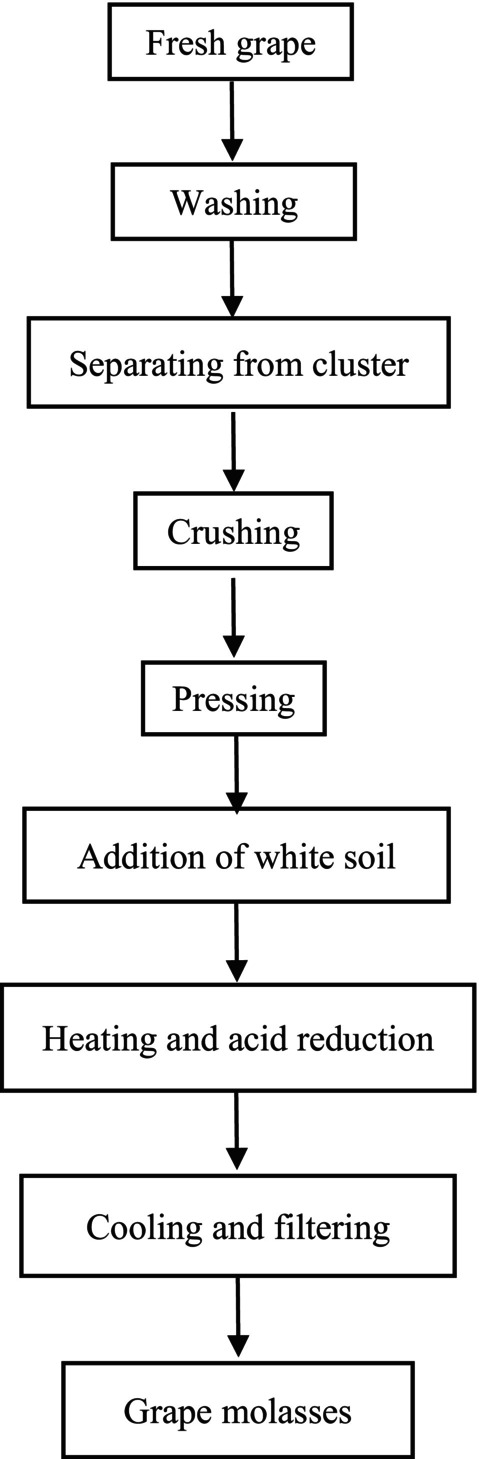
Schematic diagram of grape molasses production.

The schematic diagram for grape molasses production is illustrated in Figure 1. Grapes were washed with water at a 1 to 4 ratio for 5 min and rinsed with tap water for 15 s. Then, they were crushed using a blender (Pars Khazar, Tehran, Iran) and pressed. To separate the skin and seed, samples were passed through Whatman filter paper (No. 2), consecutively. After that, the white soil (calcium carbonate 90.0%) was added as a clarifier and heated for 10 min at 100°C. The samples remained for 24 h at room temperature (Heshmati et al., 2019) and then packaged in a dark bottle.

#### 2.2.2 Adulteration of samples

In this research, the composite models were prepared by different syrup samples. Four different sugar syrups were used in four levels of concentration (0%, 10%, 30%, and 50% according to the basis of weight) after the production process in grape molasses. High fructose corn syrup (FS), glucose syrup (GC), and sugar beet syrup (BS) were used as sugar syrups. After adding the grape molasses and syrups in the determined ratios, the samples were mixed using a magnetic stirrer for 20 min at 35°C (Tosun & Keles, 2012) and then cooled in a water bath until reaching the ambient temperature. Different tests were performed in triplicate. Table 1 shows the description and code of different samples.

**TABLE 1:** The description and code of different samples.

Sample	GM (w/v)	FS (w/v)	GS (w/v)	BS (w/v)	Appearance
T1	100	0	0	0	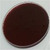
T2	0	100	0	0	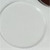
T3	90	10	0	0	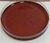
T4	70	30	0	0	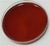
T5	50	50	0	0	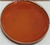
T6	0	0	100	0	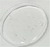
T7	90	0	10	0	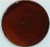
T8	70	0	30	0	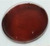
T9	50	0	50	0	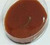
T10	0	0	0	100	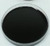
T11	90	0	0	10	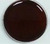
T12	70	0	0	30	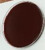
T13	50	0	0	50	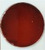

#### 2.2.3 ^13^C/ ^12^C isotope ratio analysis

After combustion of the bulk sample in elemental analyzer, generated carbon dioxide (CO_2_) passes through the system and columns. Then, they are stripped of the water in the water trap as well as CO_2_ in the purge and trap columns. The CO_2_ desorption column is heated to 110°C and the CO_2_ sample gas is released passing through a second water trap and into the isotope ratio mass spectrometry. The exact isotopic values of reference gasses to be used in this technique are determined against the internationally accepted reference materials and employed to calculate the isotopic ratios of the samples. To verify a method, a laboratory secondary standard was analyzed to check the stability of the system. The accepted δ^13^C (%) value for this standard is −27.98 ± 0.01. This standard is verified against the primary reference material of EuroVector (Elemental analyzer (EA):600 mass spectrometer‐Micromass Isoprime). Based on the reported instrumental specifications, the accepted values for δ^13^C standard deviation (1σ) were 0.1% (AOAC, 1995).

#### 2.2.4 Physicochemical properties

A digital refractometer (PAL‐22S, Atago, Japan) was used to evaluate °Brix values at 25°C (Güçlü et al., 2023). The electrical conductivity of samples was measured using a multiconductivity measuring device (HQ40d, Colorado, USA) at 25°C and it was expressed in millisiemens per centimeter (mS/cm) (AOAC, 2009). Specific gravity (SG) was determined using a pycnometer. The weights of the empty (*W*_0_) and dry pycnometers, that of the pycnometer full of distilled water (*W*_w_), and that of the pycnometer full of different samples (*W*_f_) were measured. The SG at 25°C was calculated as follows: SG = (*W*_f_–*W*_0_)/(*W*_w_ – *W*_0_). To evaluate the pH, deionized water was added to the samples and the pH was evaluated potentiometrically using a pH meter (Metrohm, Switzerland) at 25°C (Güçlü et al., 2023). The moisture content of the grape molasses samples was measured by the moisture loss on drying method using a moisture analyzer (AOAC, 2009). The samples were weighted on moisture balance and superheated until the end of the drying process time. The ash content of samples was measured based on the Association of Official Analytical Chemists (AOAC, 2009). The crucibles were heated in a furnace at 550°C for 1 h and were weighted. Then, 2 g of samples was put in the crucible. The samples were kept at 103°C until their water content evaporated, and they were burned in a furnace at 550°C until they were completely carbonized. After that, samples were cooled in the desiccator and weighed.

#### 2.2.5 5‐Hydroxymethyl furfural (HMF)

The HMF was determined quantitatively following the procedure described by Şen et al. (2020) based on a colorimetric reaction between barbituric acid, p‐toluidine, and HMF. The intensity of the red color was measured at 550 nm using a double‐beam spectrophotometer (T80+, PG Instrument, UK) at ambient temperature (Şen et al., 2020).

#### 2.2.6 Sugar content

Glucose, fructose, and sucrose content was determined according to Başaran et al. (2021). Briefly, 5 g of different pekmez samples was dissolved in 40 mL of distilled water and then 25 mL of methanol was added. Glucose, fructose, and sucrose content was extracted with water, centrifuged, filtered, and injected into an HPLC device (LC 20 AT Prominence, Shimadzu, Japan) equipped with a refractive index detector. Twenty microliters of each sample was injected into a column. Determination was performed at 30°C column temperature and 1.3 mL/min flow rate (Başaran et al., 2021).

#### 2.2.7 Rheological behavior

The rheological properties of samples were determined at 25°C by dynamic rheological tests using a rotational viscometer (Dv‐3p, Ostrich, Germany).

### 2.3 Statistical analysis

The statistical analysis was performed according to the one‐way analysis of variance (ANOVA) using IBM SPSS (version 20 for Windows). Significant differences between means were compared by Duncan's multiple‐range tests. Data were presented as mean ± standard deviation of the means.

## 3 RESULTS AND DISCUSSION

### 3.1 ^13^C/ ^12^C isotope ratio composition

The average results of δ^13^C ratios belonging to pure GM samples, FS, GS, and BS are given in Table 2. The δ^13^C values in the GM adulterated by FS, GS, and BS ranged from −23.53 to −19.99%ₒ, −25.11 to −19.70%ₒ, and −25.06 to −21.87%ₒ, respectively. The addition of FS, GS, and BS below 50% caused a statistically significant (*p* < .05) reduction in the δ^13^C value of adulterated samples. As can be seen, the isotope values of FS, GS, and BS were found to be very different from those of pure GM samples, which is due to the fact that they have different photosynthetic pathways. Other studies reported ^13^C/^12^C isotope ratio levels to lie between −24.90%ₒ (Tosun, 2014; Yücel et al., 2023) and −26.60 (Tosun, 2014). In a study that was done to find adulteration in mulberry pekmez, Tosun (2014) reported a very slight negative decrease in δ^13^C ratios depending on the syrup addition ratio (Tosun, 2014), which is in accordance with the result of the present study. A similar decrease in the δ^13^C value of honey samples adulterated by higher concentrations of beet sugar syrup was observed (Padovan et al., 2003; Tosun, 2013).

**TABLE 2:** Stable carbon isotope ratios, °Brix value, and conductivity of pure grape molasses, glucose, fructose, and beet sugar syrups.

Sample	δ^13^C (%ₒ) vs. PDB ± 1σ	°Brix	Conductivity (mS/cm)	Specific gravity (g/cm^3^)
T1	−26.61 ± 0.04i	76.93 ± 1.45d	1.80 ± 0.17e	1.55 ± 0.03d
T2	−13.42 ± 0.10a	75.87 ± 0.67ef	6.77 ± 1.07e	1.40 ± 0.01e
T3	−23.53 ± 0.08e	75.76 ± 1.26ef	0.19 ± 0.0e	1.40 ± 0.01e
T4	−23.50 ± 0.10e	75.40 ± 0.96ef	2.92 ± 0.19e	1.40 ± 0.02e
T5	−19.99 ± 0.03c	75.2 ± 0.55f	3.76 ± 0.43e	1.41 ± 0.05e
T6	−13.23 ± 0.10a	82.66 ± 0.89a	5.86 ± 0.43e	1.77 ± 0.05a
T7	−25.11 ± 0.06h	80.13 ± 0.81b	0.18 ± 0.01e	1.60 ± 0.0cd
T8	−23.93 ± 0.09f	78.86 ± 1.21bc	2.86 ± 0.46e	1.63 ± 0.07bc
T9	−19.70 ± 0.07c	78.26 ± 1.35bc	3.18 ± 0.29e	1.67 ± 0.04b
T10	−16.58 ± 0.07b	79.10 ± 0.75bc	648.83 ± 38.59a	1.81 ± 0.02a
T11	−25.06 ± 0.04h	77.74 ± 1.10cd	61.76 ± 2.98d	1.58 ± 0.03cd
T12	−24.65 ± 0.09g	77.53 ± 0.97cd	163.26 ± 6.29c	1.61 ± 0.02bc
T13	−21.87 ± 0.10d	76.50 ± 0.75de	308.60 ± 30.61b	1.68 ± 0.05b

*Note*: The means marked by the different letters are different from each other statistically (*p* < .05).

The higher changes in δ^13^C ratios of GM samples were observed in samples adulterated by FS ranging from −19.99% to −23.53%, followed by GS in the range of −19.70%ₒ to −25.11%ₒ and by BS in the range of −21.87%ₒ to −25.06%ₒ, respectively. Differences were seen in the δ^13^C values of the samples depending on the syrup addition rate. The slight differences between the obtained results and the results of previous studies of δ^13^C are related to botanical origin, geographical origin, and composition of the fruits used, and style of grape molasses production.

### 3.2 °Brix

The results of °Brix content measurement of different samples are illustrated in Table 2. GM made of different raw materials have different °Brix contents. It was found to decrease linearly with an increase in the concentration of GS, FS, and BS. In a study conducted to identify the adulteration carried out in mulberry pekmez, the °Brix content of prepared samples was decreased by increase in high fructose corn syrup ratios from 0% to 50% (Tosun, 2014). The grape variety, sugar syrup, and sugar syrup proportion affect the °Brix values (Yaman & Durakli Velioglu, 2019). Moreover, it has been reported that the °Brix content of GM samples was increased by additions, such as glucose, fructose (Güçlü et al., 2023), and sucrose syrups (Naderi‐Boldaji et al., 2018).

### 3.3 Specific gravity

The results of the specific gravity of different samples are shown in Table 2. The specific gravity of different samples ranged from 1.40 to 1.81 g/cm^3^. The addition of GS caused an increase in specific gravity, while FS reduced it. Akbulut and Bilgicli (2010) reported lower specific gravity for grape molasses (1.36 g/cm^3^), which is related to grape variety and preparation method (Akbulut & Bilgicli, 2010). The specific gravity of mulberry pekmez also increased when glucose syrup was added (Tosun, 2014). By the way, a positive correlation (*R* ^2^ > .95) was observed between ^13^C/^12^C isotope method and °Brix or specific gravity.

### 3.4 Conductivity

The results of conductivity of different samples are displayed in Table 2. The conductivity values of GM, FS, GS, and SB were 1.80, 6.77, 5.86, and 648.83 mS/cm, respectively (Türkben et al., 2016). The addition of FS, GS, and SB to the GM reduced their conductivity. The decrease observed with the addition of 10% syrup was found to be higher than the decrease observed with the addition of 50% syrup in all samples. The addition of glucose and fructose syrups to GM decreases the conductivity value since they do not contain protein, organic acids, and mineral salts and therefore reduce the total content of these components. A similar result was reported for mulberry pekmez (Tosun, 2014). Conductivity between 0.13 and 4.51 mS/cm for GM produced by different varieties of grape was also reported by Türkben et al. (2016). The slight difference in the obtained values is related to the type of fruit, its ripeness, and its features (Güçlü et al., 2023).

### 3.5 Moisture content

**FIGURE 2:**
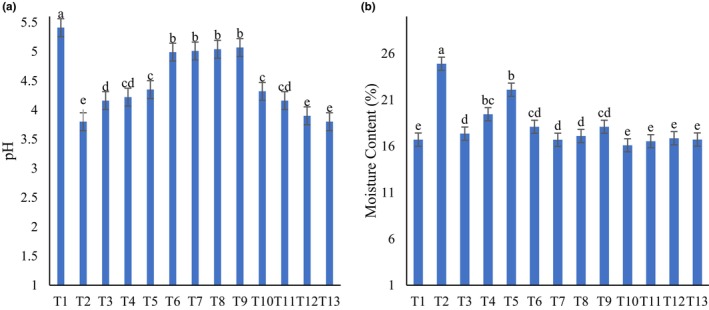
pH (a) and moisture content (b) of different samples. Different letters indicate statistically significant difference at *P* < 0.05.

The results of moisture content of different samples are illustrated in Figure 2a. The moisture content ranged from 16.1% to 22.1%. An increase in syrup concentration caused a growing trend in the moisture content of samples. As reported in previous literature, the addition of FS showed moisture content to be higher than 1.24% (Kalaycioğlu, 2023).

### 3.6 pH

The pH values of different samples are shown in Figure 2b. The pH of GM samples ranged from 3.80 to 5.41. The highest pH was observed in GM. According to the Iranian National Standards Organization (INSO), the pH level of grape molasses should be between 5.2 and 5.6 (INSO, 2012). Adding FS and GS caused an increment in pH, while BS reduced the pH. The change in pH value that occurs with the addition of sugar is an important indicator for the detection of adulteration. These results are in line with the reported values by other researchers. They reported that pH values ranged from 4.34 to 5.70 (Narkabulova & Jabborov, 2023), 3.59 to 5.23 (Türkben et al., 2016), 4.34 to 5.70 (Narkabulova & Jabborov, 2023), and 4.63 to 5.63 (Şen et al., 2020). Güçlü et al. (2023) reported a linear increase in pH values of grape molasses with the addition of GS and FS (Güçlü et al., 2023; Şimşek et al., 2004). In accordance with the present study, it has been reported that the addition of cornelian cherry to molasses for adulteration caused a significant decrease in pH values (Güçlü et al., 2023). Therefore, the trend in pH changes depends on the nature of syrups and it is not an accurate indicator to detect adulteration.

### 3.7 Ash content

**FIGURE 3:**
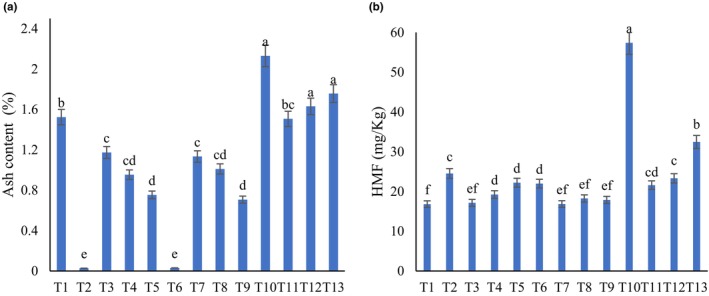
Ash content (a) and hydroxymethyl furfural (HMF, b) of different samples. Different letters indicate statistically significant difference at *P* < 0.05.

Figure 3a presents the results of ash content measurement. The ash content of GM showed a trend toward reduction with addition of both FS and GS, while there was increment observed with the addition of BS from 10% to 50%. A similar decrease in ash content was observed with the addition of GS and FS in grape, carob, fig, and mulberry syrups reported by Güçlü et al. (2023). In accordance with our results, Tosun (2014) reported that the adulteration in mulberry molasses by the addition of GS caused the reduction of ash content (Tosun, 2014).

### 3.8 Hydroxymethyl furfural

The results of the HMF content of different samples are illustrated in Figure 3b. According to the Iranian National Standards Organization (INSO), the allowed HMF level of GM should be lower than 100 mg/kg (INSO, 2012). The HMF values varied from 16.80 to 57.36 mg/kg and the addition of all sugar syrups caused an increase in HMF. Türkben et al. (2016) investigated the HMF content of GM produced by different grape cultivars, which varied between 1.00 and 762.22 mg/kg (Türkben et al., 2016).

### 3.9 Sugar content

The sugar contents of different samples are given in Table 3. In the present study, the amount of the fructose content in GM ranged from 0.79% to 55.33%. The addition of FS caused an increase in fructose content of GM, while the addition of GS and BS reduced the fructose content. Sucrose content in GM containing GS and FS was decreased. Generally, the change in content of each additive to GM depends on their origin and features. According to the fructose to glucose (F/G) ratio reported by Kalaycioğlu (2023), adding GS and SB did not affect the F/G ratio of the GM, but the effect of FS on it was significant (Kalaycioğlu, 2023).

**TABLE 3:** The content of fructose, glucose, sucrose, and total sugar of different samples.

Sample	Fructose (%)	Glucose (%)	Sucrose (%)	F/G	Total sugar (%)
T1	49.60 ± 0.65c	50.57 ± 1.27a	0.26 ± 0.04e	1.01 ± 0.03d	66.85 ± 1.36e
T2	55.33 ± 0.25a	29.49 ± 0.52g	–	1.89 ± 0.04a	96.51 ± 0.55a
T3	50.29 ± 0.29bc	42.93 ± 0.66c	0.20 ± 0.01e	1.17 ± 0.02c	66.93 ± 0.55e
T4	51.71 ± 0.33b	35.9 ± 1.41f	0.20 ± 0.02e	1.44 ± 0.08bc	72.40 ± 0.62c
T5	52.26 ± 0.27b	31.33 ± 0.40g	0.17 ± 0.02e	1.61 ± 0.04b	79.50 ± 0.75b
T6	0.79 ± 0.92k	41.83 ± 0.15d	–	0.01 ± 0.00	41.80 ± 0.62j
T7	39.76 ± 0.18e	48.66 ± 0.45ab	0.20 ± 0.02e	0.79 ± 0.01g	60.97 ± 0.72g
T8	32.56 ± 0.77h	44.90 ± 0.80c	0.21 ± 0.01e	0.73 ± 0.01g	54.93 ± 0.65f
T9	29.30 ± 0.85i	43.83 ± 0.32c	0.18 ± 0.02e	0.65 ± 0.03h	50.53 ± 0.66i
T10	17.30 ± 1.35j	20.39 ± 0.42h	60.53 ± 0.977a	0.88 ± 0.03f	80.83 ± 0.64b
T11	44.8 ± 0.27cd	47.46 ± 1.32b	5.78 ± 0.33d	0.95 ± 0.01de	63.70 ± 0.45f
T12	37.63 ± 0.93f	43.13 ± 1.15c	16.02 ± 1.41c	0.88 ± 0.02f	68.63 ± 0.41d
T13	34.96 ± 0.80g	37.26 ± 0.56e	25.42 ± 1.08b	0.92 ± 0.02ef	74.73 ± 0.47c

### 3.10 Rheological properties

**FIGURE 4:**
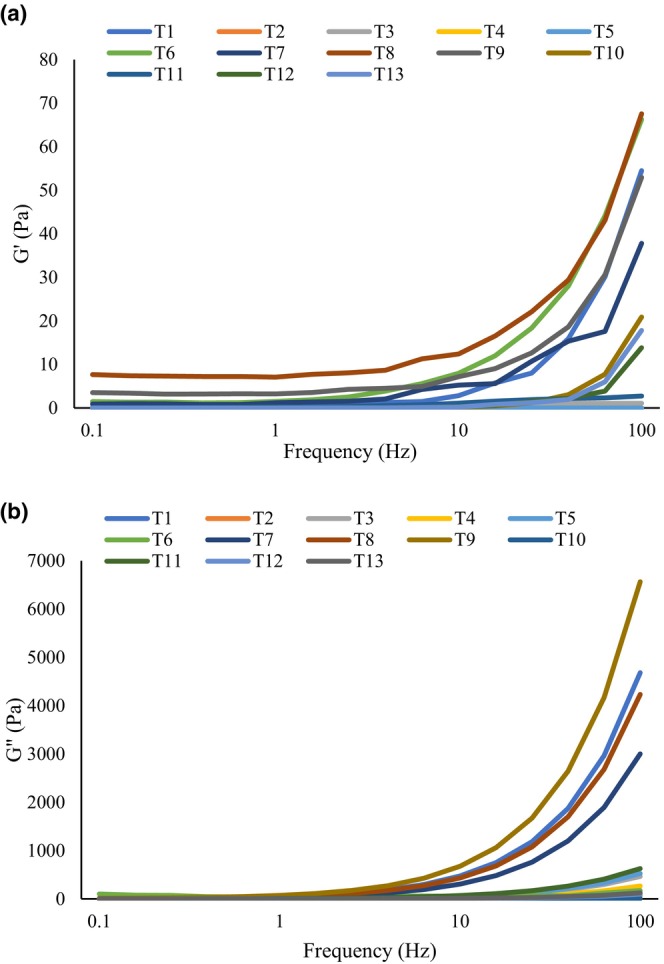
The change in (a) storage modulus G' and (b) loss modulus G" of samples.

The change in storage modulus G' and loss modulus G" of different samples as a function of frequency is illustrated in Figure 4. The magnitudes of G' and G" increase with an increase in frequency representing the viscoelastic behavior of samples. At low frequencies, G' > G" elastic behavior is obviously seen, while at intermediate frequencies viscose behavior is seen. These results are in agreement with those reported by Tang et al. (2017) who reported typical elastic behavior for grape molasses (Tang et al., 2017).

## 4 CONCLUSION

Grape molasses is a highly nutritional product that is used due to its health benefits and nutritional value. Adulteration of grape molasses has an important effect on producers and consumers. The current study was the first attempt to investigate the application of the ^13^C/^12^C isotope ratio to determine the adulteration of Iranian grape molasses. The addition of any type of adulterant, such as glucose syrup, fructose syrup, and beet sugar syrups, causes a change in ^13^C/^12^C isotope ratio values. A positive correlation was observed between chemical results and ^13^C/^12^C isotope ratio values. The process of changes in physicochemical parameters by adding glucose, fructose, or molasses syrups can be an increasing or decreasing trend. In addition to tests, the measurement of physicochemical parameters takes time and requires a lot of chemical reagents. While, by using the ^13^C/^12^C isotope ratio, it is possible to find out the adulteration of grape molasses faster and using fewer chemical reagents. The results of this research suggest the use of ^13^C/^12^C isotope ratio as an accurate and alternative method to detect the adulteration of grape molasses.

## AUTHOR CONTRIBUTIONS

**Vahid Jamali:** Conceptualization (equal); data curation (equal); investigation (equal); methodology (equal); software (equal); writing – original draft (equal); writing – review and editing (equal). **Aryou Emamifar:** Formal analysis (equal); methodology (equal); project administration (equal); resources (equal); software (equal); supervision (equal); writing – original draft (equal). **Hadi Beiginejad:** Conceptualization (equal); data curation (equal); formal analysis (equal); validation (equal); visualization (equal); writing – original draft (equal). **Mohammad Moradi:** Data curation (equal); funding acquisition (equal); methodology (equal); project administration (equal); validation (equal). **Mousa Rasouli:** Conceptualization (equal); data curation (equal); software (equal); writing – original draft (equal); writing – review and editing (equal).

## FUNDING INFORMATION

None.

## CONFLICT OF INTEREST STATEMENT

The authors have declared no conflict of interest.

## ETHICS STATEMENT

This article does not contain any studies with human or animal subjects.

## Supporting information


Figure S1

## Data Availability

Data will be made available on request.
