# Supplementary material for: Detection of adulteration in Iranian grape molasses added glucose/fructose/sugar beet syrups with 13C/ 12C isotope ratio analysis method
Source: Food Sci Nutr. 2024 Sep 10;12(10):8432–40. doi: 10.1002/fsn3.4259 (PMC11521713; doi:10.1002/fsn3.4259)

**Supplementary Fig**

**Fig S1.** A typical chromatogram of *^13^C/^12^C* for grape molasses sample. The traces show signal intensity in mV of the simultaneously collected masses (m/z) of 44, 45 and 46 amu. The acquisition time is expressed at seconds (s).


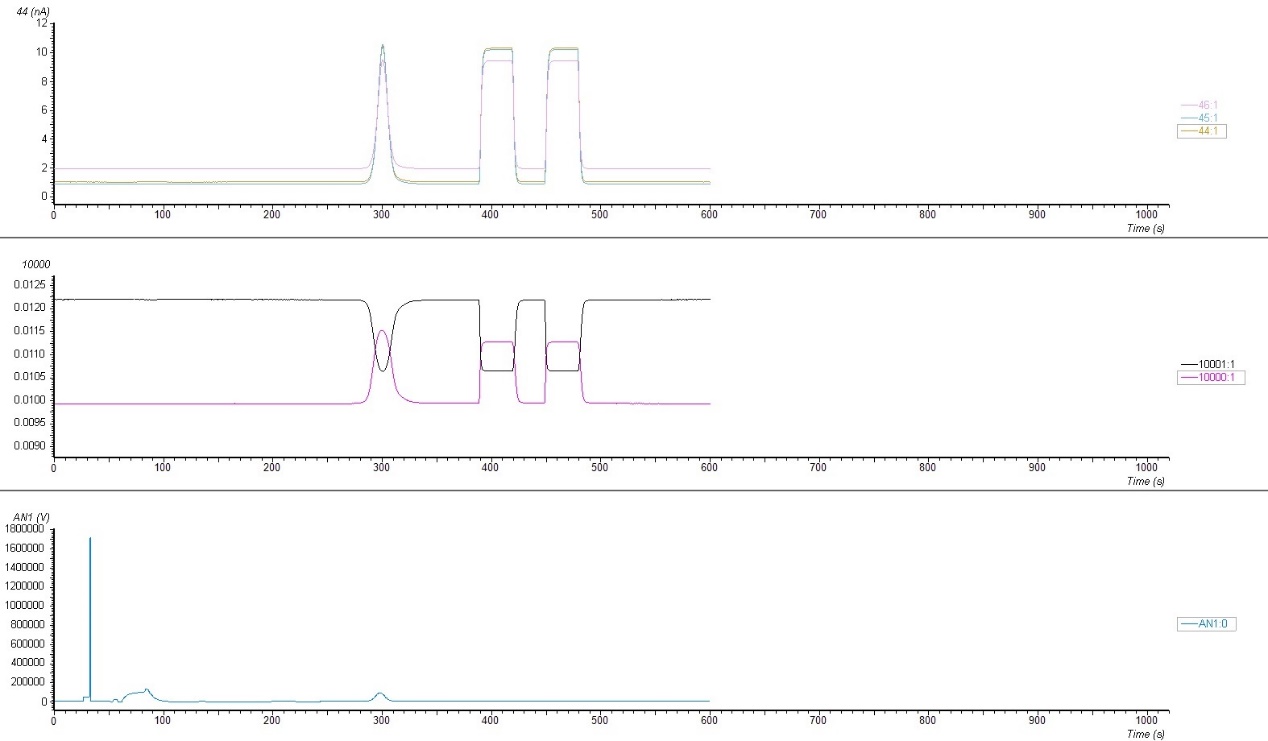

Supplement: Supplementary file 1 — Figure S1 [file FSN3-12-8432-s001.docx]
